# Safety and wellbeing as spatial capacities: An analysis from two ethnographic studies in primary care and palliative care contexts

**DOI:** 10.1016/j.healthplace.2018.08.020

**Published:** 2018-11

**Authors:** Suzanne Grant, Aileen Collier

**Affiliations:** a Population Health and Genomics, School of Medicine, University of Dundee, The Mackenzie Building, Dundee DD2 4BF, UK; b School of Nursing, University of Auckland, Auckland, New Zealand

**Keywords:** Patient safety, Wellbeing, Space, Primary care, Palliative care

## Abstract

Patient safety and quality of care are increasing concerns for healthcare internationally. This paper examines the spatial achievement of safety and wellbeing by healthcare staff, patients and their carers within UK primary care and Australian palliative care contexts. Two key socio-spatial modes of safety and wellbeing were found across these healthcare contexts. The technical mode was spatially managed by staff and driven by formal approaches to safety with a limited focus on wellbeing. In contrast, the relational mode was driven by attentiveness to the wellbeing and spatial engagement of staff, patients and carers that drew on informal elements of safety. Both modes extended across public, private, biomedical and administrative spaces, with technical and relational safety-wellbeing configurations often inhabiting the same spaces. Differences also existed across primary and palliative care contexts that reflected the unique pressures present within each context, and the ability of people and places to adapt to these demands. In the context of increasing workloads in healthcare internationally, this study highlights the benefits of attending as much to the relational dimensions of safety and quality of care as to the technical ones through increased focus on the safety and wellbeing of healthcare staff, patients and carers within and beyond traditional sites of care.

## Highlights

• Two key modes of safety and wellbeing exist across primary and palliative care contexts.
• The technical mode is spatially managed by staff and driven by formal approaches to patient safety.
• The relational mode is driven by attentiveness to the wellbeing and spatial engagement of staff, patients and carers.
• Both technical and relational modes extend across public, private, biomedical and administrative spaces.
• Future improvement initiatives should focus on both technical and relational modes of safety and wellbeing.

## 1 Introduction

Over the past decade, there has been a trend in the social sciences towards what has been termed a ‘spatial turn’ (Warf and Arias, 2008). In this work, space is understood as dynamically produced and reproduced in pre-discursive, discursive and practical ways (Massey, 2005, Massey, 2013, Thrift, 2008). However, rather than exclude the material environment, this conceptualisation includes the material as an essential actor in the production of space (Munn, 1996). Corsín-Jiménez (2003) writes that this understanding of space conceptualises agency as distributed across people and the material world as *spatial capacities*. This agency-centred, relational understanding of space has resulted in renewed interest in the spatial qualities of healthcare organisation and delivery (Billo and Mountz, 2015, Malpas, 1999, Street and Coleman, 2012). While earlier studies have generally focused on hospitals as formal, bounded spaces of biomedical control (Coser, 1962, Goffman, 1961), more recent research has emphasised the informal complexities of healthcare spaces beyond hospital environments (Seamon and Sowers, 2008, Wilson, 2003). Two dimensions of healthcare where space has been of increasing concern are in the fields of patient safety and wellbeing.

Patient safety and quality of care are increasing concerns for healthcare internationally (The Health Foundation, 2014, Institute of Medicine (IOM), 1999). One dimension of healthcare quality that has received increasing attention is wellbeing. Defined as “an optimal state for an individual, community, society and the world as a whole” (Mathews and Izquierdo, 2009: 5), dominant approaches to quality improvement have often applied pre-prescribed standards of care to measure and manage wellbeing based on pre-defined, individually-focused quality indicators. This approach is also present in the field of patient safety, where significant attention has focused on eliminating adverse events and applying a compliance-based approach to safe practice based on pre-prescribed protocols and guidelines (Reason, 1990, Vincent et al., 2013). However, more recent research has examined how safety and wellbeing are achieved spatially in everyday practice (Atkinson et al., 2012, Iedema et al., 2010). While this research has developed more nuanced understandings of safety or wellbeing amongst healthcare staff, how these concepts interrelate and the wider role of non-clinical staff, patients and their carers (i.e. family members, partners, friends) remains less well understood. This paper examines the spatial achievement of safety and wellbeing by healthcare staff, patients and carers across two healthcare contexts: UK primary care and Australian palliative care.

## 2 Background

More recent approaches to understanding healthcare spaces have emphasised their informal qualities and how they incorporate different modes of socio-spatial ordering (e.g. biomedical, emotional) (Lefebvre, 1991, Street and Coleman, 2012). These have been shown to continually realign to create dynamic socio-spatial configurations between human actors and their wider spatial environments (Law and Mol, 2001, White et al., 2012). The following section examines how space has been examined within the fields of patient safety and wellbeing.

### 2.1 Situating patient safety in healthcare organisations

Dominant approaches to patient safety improvement have historically involved the application of ‘measure-and-manage’ approaches (e.g. Significant Event Analysis) to formally identify features of a workplace or clinical process where performance can be improved through strict adherence to formal guidelines (Reason, 1990, The Health Foundation, 2011). While these approaches have led to significant improvements, the complex interrelationship between people and their wider workplace environments mean that cause and effect are not always linked in a predictable manner (Iedema et al., 2006, Patterson, 2008). Furthermore, while adverse events are not uncommon, things go right the majority of the time (Hollnagel, 2014, Mesman, 2011). Complementary ways of understanding and improving safety have since emerged that draw attention to the positive dimensions of safety and the informal adjustments made by healthcare teams when faced with less predictable risks (Grant et al., 2017, Grant et al., 2016, Iedema, 2009). Thus, rather than focus on the elimination of error, this research focuses on how risk is mitigated and safety achieved through informal knowledge and collaboration between professionals, patients and carers (Braithwaite et al., 2015, Waring et al., 2015).

One focus of this research has been on the relationship between healthcare professionals and their workplace spaces, and how factors such as spatial layout and equipment impact on the delivery of safe patient care. Hor et al. (2014), for example, found that open ward spaces in an Australian intensive care unit were both conducive to safety due to the opportunities that they presented for informal communication and risky due to increased interruptions. In order to safely manage these risks, staff created temporary *protected spaces* through the use of curtains and signs. Similarly, Iedema et al. (2010) highlighted the central role of informal *liminal spaces* (e.g. corridors, stairwells) in a hospital outpatient clinic and the opportunities that these spaces provided for staff to communicate key safety information in an impromptu manner. Healthcare workers were therefore continually negotiating safety informally, with less visible spaces crucial to the ongoing mitigation of risk. While this more recent body of work has been important for understanding how clinicians achieve safety within single-site hospital spaces, the spatial dimensions of safety beyond acute care (Grant et al. 2016) and the role of non-clinical administrative staff (Grant et al., 2017; Swinglehurst et al., 2011), patients and their carers (Collier and Wyer, 2015, Hor et al., 2013) remain less well understood.

### 2.2 Wellbeing and place in healthcare

Over the past decade, there has also been renewed interest in wellbeing within healthcare policy and practice, with dominant approaches focusing on the measurement of individual wellbeing according to pre-defined standards of care (Graham, 2012, Mathews and Izquierdo, 2009). Ferraro and Sarmiento Barletti (2016) write that this has resulted in relatively little attention being paid to the role of context in shaping understandings and practices of wellbeing (see also Fischer, 2014; Thin, 2008). In response to this, more recent research has examined how people understand and create place through everyday relationships, practices, emotions and memory, with wellbeing conceptualised as both context-specific and relational (Atkinson et al., 2012, Corsín-Jiménez, 2008). Midwifery scholars, for example, have examined the role of place on how midwives conduct their work, with home or home-like settings providing women with a greater sense of control and physical and emotional comfort than hospital settings, where they feel more like visitors (McCourt et al., 2016, Walsh, 2006).

Two healthcare contexts where research on safety, wellbeing and space have been more limited are primary care and palliative care. Primary care is the first point of care for most patients, the principal point of continuing care, and the place from which specialist care is usually coordinated (Starfield et al., 2005). In contrast, palliative care seeks to improve the quality of life for patients facing a life-threatening illness through the prevention and relief of suffering (World Health Organisation (WHO), 2012). Crosscutting primary, community and acute care settings, palliative care involves the early identification, assessment and treatment of pain alongside the physical, psychosocial and spiritual needs of patients and their carers. Alongside these key differences, there are also important similarities between these types of healthcare delivery including that they are delivered across multiple spaces that crosscut healthcare organisations, the community and people's homes, they engage with diverse patient populations, they aim to be person-centred rather than disease-focused, and they involve multiple actors (i.e. clinicians, administrators, patients and carers) (Olesen et al., 2000). In terms of national context, both UK and Australian healthcare systems face similar challenges through increasing numbers of frail older people with multimorbidity, increased workforce pressures, and inequities in health and access to services (Commonwealth of Australia, 2010a, Barnett et al., 2012). Current healthcare policy across these two national contexts aims to enhance the capacity of primary care to assist older people, including those with palliative care needs, to remain in their own homes for as long as possible (Commonwealth of Australia, 2010b, Guthrie et al., 2018). These policies place general practice at the centre of care provision to meet both the current and anticipated growth in need, with a recent report asserting that UK general practice is in crisis (Baird et al., 2016). Similarly, a recent Australian Productivity Commission report emphasised significant shortfalls in Australian palliative care and pressures on primary care and other generalist palliative care providers to meet the needs of older Australians (Productivity Commission, 2017). The aim of this paper is to ethnographically examine the interrelationship between safety, wellbeing and space across UK primary care (Study 1) and Australian palliative care (Study 2) within the context of increasing pressures on healthcare organisation and delivery across these two contexts.

## 3 Methods

Study 1 was a multi-site ethnographic study conducted in UK primary care from January 2011-April 2014 that examined the ways in which safety and quality in high-volume organisational routines (e.g. repeat prescribing) were achieved spatially and temporally across different general practice settings. Study 2 was a video reflexive ethnographic (VRE) study conducted in Australian palliative care from May 2010–September 2011 that examined the spaces where people with life-limiting illnesses received care and how these contributed to the safety and quality of care delivered. Table 1 summarises the study design, timeframe, setting, participants, and methods of data collection and analysis across each study.

**Table 1:** Study design, setting, data collection and data analysis for Studies 1 and 2.

**Study**	**Study design**	**Study setting and participants**	**Data collection**	**Data analysis**
Study 1	Multi-site ethnography combining non-participant observation of the everyday working practices of team members with interviews and documentary analysis to develop an in-depth understanding of patient safety and quality of care within each general practice.	Eight general practices in NHS England and NHS Scotland were purposively selected on the basis of their size (smaller ~4,000 patients or larger ~9,000 patients), the socioeconomic deprivation of the population served (affluent, mixed or deprived), and location (urban or rural).	Data collection took place in two phases. A long-term ethnographic study was conducted in Practices 1–4 over a 24-month period followed by focused fieldwork in Practices 5–8 over one-week periods examining specific high-volume organisational routines (e.g. repeat prescribing, laboratory test results handling).	Analysis was informed by the constant-comparative method (Charmaz, 2014). Both fieldnotes and interviews were annotated with observational and theoretical notes as fieldwork progressed and were shared between the research team. The researcher read the interview transcripts to become familiar with the data. Preliminary themes were identified through scrutiny of initial transcripts and a coding framework was subsequently developed that was embedded in the data collected. Data within each practice setting were firstly examined within their own context before being compared across contexts to develop higher-level concepts and to identify any differences and/or similarities.
			The researcher (trained in social anthropology) undertook 1,787 hours of ethnographic fieldwork. Informed consent was obtained from each practice team member prior to fieldwork commencing. Fieldwork was undertaken with clinical, managerial and administrative staff during normal working hours in reception areas, back-offices, consulting rooms, administrative offices, meeting rooms, coffee rooms and corridors. Detailed handwritten fieldnotes were made in full view of informants, and later transcribed for coding. Informal explanations were elicited from staff as they conducted their everyday work. These descriptions were recorded with permission in the researcher's fieldwork diary and later transcribed and coded. Documentary analysis of relevant written protocols (where available) and patient information leaflets from each practice was also conducted.	
		Within each practice, the everyday working practices of clinical, managerial and administrative staff were observed.		
			62 semi-structured interviews were conducted with GPs, practice nurses, practice managers and receptionists known from observation to be involved in key organisational routines in their practices. Informed consent was obtained from each team member prior to interviews commencing.	
			Ethical approval was obtained from the local Medical Research Ethics Committee (REC) [name of committee removed for double blind peer reviewing].	
Study 2	Multi-site video reflexive ethnographic (VRE) approach.	There were two main fieldsites: 1) a palliative care day hospital (PCDH) located within a 72-bed sub-acute hospital; and 2) a large, acute metropolitan hospital.	Phase 1 of fieldwork in the PCDH took place every week over a four-month period (18 days in total). Phase 2 in the acute hospital took place over a 68-day period. The researcher shadowed patients and healthcare workers to better understand everyday practices and experiences.	Data analysis proceeded simultaneously with data collection. Consistent with VRE methods, participants guided the data analysis. First, the researcher organised the data into prominent and consistent themes across all data sets. These emerging themes were then edited into video clips considered representative of these key themes to show back to participants as part of a continuous process of immediate critique and comparative analysis. Video reflexive focus group sessions were also video-recorded and used to further refine themes.
	VRE involves negotiated videoing between a researcher and participants of everyday practices and/or experiences and the co-interpretation of the resulting footage with participants to make sense of the visual data. VRE requires participants to: feature in and/or gather visual data (V); interpret the data by monitoring and affecting events, conducts and contexts in situ (R); and use different research methods to suspend and understand practices and experiences in situ (E). VRE helps make visible not only the views and feelings of participants but also the spatial aspects of such views.			
		There were three categories of participants: (1) patients living with a life-limiting illness; (2) carer(s) of the patient (nominated by the patient); and (3) clinicians identified by the patient.	70 semi-structured interviews were conducted in total. Participants included 29 patients, 5 family members, 36 healthcare workers (nurses, doctors, allied health and administrative staff). Six of the 29 patients and family members were followed over a period of time and provided with participant-generated footage and accounts. The researcher also followed patients and carers back into other healthcare settings including the specialist palliative care unit, residential aged care and their own homes.	
			Video reflexive focus group sessions ranged from 6–28 clinician participants and represented the following specialties: palliative care; respiratory; surgical; renal, and medical oncology/haematology. In addition, sessions with larger hospital audiences (up to 100 attendees) were also conducted. In all sessions the researcher asked participants: “If you were to make visible to clinicians what is most important to your care what would you want them to see and to know?”	
			Institutional ethical approval was granted from both university and local healthcare institutional human research ethics committees [names of committees removed for double blind peer reviewing].	

### 3.1 Theoretical framework and cross-study analysis

This paper draws together context-specific examinations of the interplay between safety, wellbeing and space in primary and palliative care. Across both studies, fieldwork was informed by literature on positive approaches to healthcare safety (Hollnagel, 2014, Iedema, 2009), practice-based theories of space and social action (Corsín-Jiménez, 2003, Massey, 2005); the role of space in the inter-professional achievement of safety (Hor et al., 2014, Iedema et al., 2010, Mesman, 2009), and the role of patients and their carers in the achievement of safety (Collier and Wyer 2015). This provided a significant opportunity to study the similarities and differences within and across these different contexts. Integrating data analysis across both studies revealed the importance of wellbeing in understanding how safety was achieved spatially. Subsequent analyses across both studies have therefore also drawn on anthropological studies of wellbeing (Corsín-Jiménez, 2008, Fischer, 2014, Mathews and Izquierdo, 2009) and the interrelationship between wellbeing and place (Atkinson et al., 2012, Ferraro and Sarmiento Barletti, 2016). In order to ensure consistency and comparability across concepts and definitions, both researchers exchanged and discussed relevant data, which included interview and fieldnote extracts.

## 4 Findings

Safety and wellbeing were spatially enacted in two key ways across primary and palliative care contexts. The first was the ‘technical’ mode and the second was the ‘relational’ mode. The following sections examine the key characteristics of these two modes of safety and wellbeing as everyday socio-spatial configurations across primary and palliative care contexts.

### 4.1 The technical mode of safety and wellbeing

A technical mode of safety and wellbeing was enacted by healthcare staff when time was limited or the volume of work was particularly high. As a result, the technical mode was professionally driven and spatially managed by staff, with significant focus on formal safety rules and procedures and more limited attention paid to the wellbeing of staff, patients and carers. This following sub-sections examine different ways in which technical safety-wellbeing configurations were enacted within primary and palliative care contexts.

#### 4.1.1 Technical safety-wellbeing configurations in primary care

In Study 1, general practitioners (GPs) were under significant pressure to ensure that each patient consultation was conducted within the allocated 10-min timeslot, with individual GPs adopting different approaches to manage this. One of the GPs in Practice 2, for example, explained that he ensured that his consultations did not digress to *“chit chat and other stuff*” (GP7, Practice 2) by keeping the windows of his consulting room open during surgeries so that patients were not encouraged to remain any longer than necessary:

*I always keep a couple of windows open so that there is a bit of personal discomfort. I learned that in the practice that I trained in […] The patients never hang around for long.* (Study 1, Practice 2, GP7, consulting room, fieldnotes, 23.11.11)

In this example, the GP retained control of the ambiance of the private, biomedical space of his consulting room to ensure that the concerns of the patient were distilled to the key clinical reason for their visit. Safety was therefore framed in terms of the rapid, efficient filtering and processing of single patient concerns, with the temporary disruption of their wellbeing via the cold consulting room space facilitating this process.

Across the eight general practices in Study 1, the high-volume nature of key organisational routines (e.g. repeat prescribing, test results handling) meant that practices were each required to develop systems to maximise efficiency whilst ensuring that safety was maintained across their administrative spaces. For example, all Practice 7 patients were required to provide three forms of identification (i.e. their name, address, date of birth) when collecting their repeat prescription from the front desk. Despite this system formalising communication between receptionists and patients, who often met on a bimonthly basis when the patient came into the practice to collection their repeat prescription, it was considered a necessary safety intervention by the practice manager:

*Although it was a bit of a struggle to start with, the patients must, and do, tell us their name, address and date of birth […]. We make sure the patients tell staff this and if I find out that staff are letting patients off then woe betide them, because it is essential to ensuring that the right drug goes to the right person.* (Study 1, Practice 7, Practice Manager, main reception, fieldnotes, 24.02.14)

Once the correct prescription had been identified by staff, patients were then required to sign and date a slip of paper containing details of the prescription as a record of its collection. This additional formal step was also considered necessary due to the challenging patient population:

*This is a safety net for us […] We tend to have a lot of patients who are unsure about a lot of things because they are elderly or have learning difficulties and that's reflected in the system we have in place.* (Study 1, Practice 7, Receptionist 2)

While this formal safety work provided identifiable evidence of who had collected their prescription and when, it placed significant pressure on the front desk area of the practice as it reduced the speed at which patients could be attended to, thus creating long queues and reducing patient privacy when talking with the receptionist. It also minimised informal communication between staff and patients due to the need to focus on formal safety procedures. In order to manage the queue and ensure a degree of privacy for patients, the practice manager had introduced bollards and ropes to the main reception area. However, patients at the front desk could still be overheard by those waiting in the queue within the public administrative space of the front desk area. Despite these issues, the technical achievement of safety was considered too important to compromise and the system was maintained.

Within UK general practice, the technical mode of safety and wellbeing comprising efficiency, formal procedures and consistency of care was thus used as an ordering device by staff across biomedical, administrative, public and private practice spaces. Driven by formal approaches to patient safety, the wellbeing of both staff and patients was deprioritised, with minimal attention paid to informal communication and the socio-spatial autonomy of patients.

#### 4.1.2 Technical safety-wellbeing configurations in palliative care

In the Australian palliative care context, the technical mode was also closely aligned to formal safety rules and procedures, with hospital safety policies frequently cited by staff as a reason for not granting patient requests:

*The staff specialist (Palliative Care) said: “I’ve just been to see Mrs [name of patient]. She doesn’t want the curtains open because there's a man opposite, but having the curtains around seems to be upsetting her. There are two areas where there are two women sharing with two men and so I asked the Acting Nurse Unit Manager (ANUM) why he couldn’t change them over so that the men were sharing and the women sharing”. The staff specialist expressed her frustration and disappointment with the response: “The ANUM said that even if they move her she’ll find something else to complain about. I can see she's been labelled, but the ANUM said, ”It's just how they come in and it's hospital policy”*. (Study 2, Specialist Palliative Care Team weekly meeting, acute hospital, fieldnotes, 28.03.11)

In this example, hospital policy was used as a device for staff to order the public biomedical space of the hospital ward. Despite acknowledging the patient's reasons for requesting the move, including the negative impact that the mixed ward space was having on her wellbeing, hospital policy was considered a legitimate reason for maintaining the status quo. In other situations, clinicians would impose their own limits on the time that they spent with patients and their families as a way of minimising interaction when they themselves felt emotionally unsafe:

*As an intern, I used to dread having to certify a death […] My first rotation was just nuts, it was dreadful, because there would be deaths and you’d have to go up to a ward where you didn’t know the nursing staff, the lights were sometimes right off. You know, you knew no-one. There's this dead body and you’ve got a torch and you shine another torch in their eyes and have a listen to their chest and out you go and write on the bits of paper or there's still a whole lot of family around and it's like well you have no relationship with this family and you’ve never met them before and you’re fearful. The reality is you probably can’t help them very much and I certainly didn’t know how I could and didn’t know what to say and I think I just went in and out as quickly as possible as [a means of] avoidance.* (Study 2, SSI, staff specialist (Radiation Oncology), acute hospital, fieldnotes, 25.03.11)

In this example the intern doctor's feelings of fear and unsafety were compounded by him having to carry out a certification of the death at night when there were no senior medical staff in the proximity. The doctor knew that bereaved families had expressed needs at the time of death. However, he felt isolated and without the skills and support to respond appropriately and so removed himself from the situation as soon as possible in order to feel safe. As a result, rather than being a relational encounter with a bereaved family, the certification of the death was reduced to a technical task performed on a dead body in an unfamiliar space.

Within the emotionally demanding palliative care context, technical safety was also used to order both public and private biomedical spaces through attentiveness to formal rules and procedures. In this context, staff and patient wellbeing were often deprioritized when staffing levels were low or when staff felt emotionally unsafe.

### 4.2 The relational mode of safety and wellbeing

Both primary and palliative care staff also engaged in an alternative relational mode of safety and wellbeing that was grounded in affective relationships between staff, patients and carers across public, private, biomedical and administrative spaces. The following sections examine how this was enacted within each context.

#### 4.2.1 Relational safety-wellbeing configurations in primary care

In Study 1, doctors in all of the practices had high numbers of older patients with chronic conditions (e.g. diabetes, asthma, epilepsy) whom then saw regularly as part of National Health Service (NHS) chronic disease management initiatives. The following example illustrates how a GP in Practice 1 engaged with both the safety and wellbeing of an elderly patient whom he met regularly within the private space of his consulting room:

*An elderly female patient of approximately 70 years of age entered GP2's consulting room during the morning surgery and sat down on one of the two chairs positioned to the side of his desk. GP2 had told me before she arrived that her husband had recently died of a heart attack and he began the consultation by asking her how she was doing. She replied that she “wasn’t doing great” as her son had recently moved out of her home before going on to tell him about her recent appointment at the renal clinic in the local hospital: “I was told that my kidney function had settled, that it wasn’t going up and down like it used to do”. GP2 then asked how she was managing on her tablets. The patient replied. “You have me down as 5 mg, which is 2.5 mg twice a day. You had me on that before Jack [her husband] died, but it's not that, it's 5 mg twice a day now”. GP2 looked at his computer screen and replied “Yes, we increased it after then. You’re right that it's still needing updated, so I’ll change that now”. He then turned his computer screen so that the patient could see what he was doing before going into the repeat prescribing system to update the dosage. GP2 then went on to tell the patient that he was retiring in the coming weeks. The patient replied “That's two bereavements in one go. This is not a good time to tell me”. She then started talking about how she had been a patient of the practice for over 26 years and pointing to the four old pictures of the practice that GP2 had on his wall from its previous location in a Victorian house nearby as she spoke about how it had been “back then”. While she was talking, the patient started crying and GP2 handed her a tissue. When she left, GP2 told me that he “felt guilty” for telling her his retirement plans but that it was best that he told her in his own space than for it to be conveyed by “someone at reception”. “I’ll write her emotional state in her notes for when someone new sees her”* (Study 1, Practice 1, GP consulting room, fieldnotes, 24.01.12).

In this example, the GP acknowledged the patient's physical and emotional wellbeing before, during and after the consultation. Despite her emotional vulnerability, the patient felt safe enough in the private biomedical space of his consulting room to correct the GP on her medication dosage and inform him of the emotional impact that his retirement was having on her.

Across all eight practices in Study 1, receptionists played a key role in processing patients’ medication requests, with this often involving them making special cases for particular patients based on both their knowledge of the patient and their medication, the safe limits of ‘the system’, and the most appropriate practice spaces to attend to that patient's needs:

*At 10:40 a.m., a middle-aged woman approached the reception front desk and it was clear that Receptionist 2 already knew her as they spoke about their grown up children. The patient then went on to mention her asthma and how she had been feeling “really wheezy” lately and if she could have an additional Salbutamol inhaler (on top of her regular repeat prescription) to control her symptoms. The receptionist said that she would “see what [she] could do” and asked the patient to take a seat on one of the chairs next to the front desk while she spoke to the duty doctor. The chairs had been positioned there specifically for situations like these, with magazines placed on a small table nearby and radio music in the background as a form of “sound-proofing” (Practice Manager) to create privacy for those at the desk. Receptionist 2 then made her way down the corridor and waited outside the duty doctor's consulting room for the patient that he was seeing to leave. After the patient had left, Receptionist 2 approached the duty doctor, showed him the patient's prescription request slip and the reason for the request. The doctor agreed to authorise the additional inhaler on the condition that the patient made a further appointment with him for a check-up. On her way back down the corridor, Receptionist 2 explained: “I know a lot of our patients on first-name terms and I try to make them feel welcome when they come to the front desk […] I think you can relay things better to the doctor when you understand where the patient is coming from and they trust you.”.* (Study 1, Practice 4, front desk, 20.04.12)

In these examples, relational configurations of safety and wellbeing were based on patient involvement in the informal safety work of general practice clinical and administrative staff. These informal relationships often extended over long periods of time and took place within familiar practice spaces that extended beyond the formal biomedical space of the consulting room into the public administrative space of the front desk and the liminal spaces of the practice corridors.

#### 4.2.2 Relational safety-wellbeing configurations in palliative care

In the palliative care setting, safety and wellbeing were often attended to by staff, patients and carers within private biomedical spaces:

*The palliative care nurse (PCN) and I entered the four-bed bay to see an elderly patient who was in the bed in the corner beside the window. The curtain between her and the next patient was closed. It was evident that she was dying. The patient's daughter sat on the table beside the window and the PCN sat down beside the patient and asked how she was. There was what I would describe as a tender directiveness in their interaction. The nurse sprayed some water gently into the lady's mouth with a plastic spray bottle as she gently stroked her head. It was obvious that the water was appreciated. At the same time, the patient appeared agitated as she tried to remove the bed covers. The PCN proposed that the patient may be too hot and so removed the blanket. The patient's daughter expressed that she thought her mother could be itchy. “If she is itchy then we can use this” the PCN replied, reaching for the moisturizer. I watched as she dispensed some into her hand and handed the container across the bed to the patient's daughter to do the same. In mirror image they stood at either side of the bed rubbing the cream gently into the lady's arms. I was touched as I watched the actions of the patient's daughter and the nurse in what appeared to be a moment of intimacy.* (Study 2, acute hospital, fieldnotes, 28.01.11)

Through her attentiveness to the physical body of the patient, the nurse cared for the whole person in a way that did not separate physical and emotional care. The nurse took account of these elements by respectfully talking to the patient despite the fact that she was moribund. She was also open to the suggestion by the patient's daughter that her mother may be itchy and responded with humility and openness in what became a temporarily shared private space between the dying patient, her daughter and the nurse.

In Study 2, staff, in patients and their families would frequently interact around the communal lounge and kitchen spaces of the day care hospital attached to the in-patient unit as pre-defined places of wellbeing. Patients would spend most of their time around a communal table partaking in individual or group tasks (e.g. painting, knitting, board games) facilitated by the diversional therapist and supported by specialist nurses, senior doctors and allied health staff in the day room. The spatial interconnection between the in-patient and day care units meant that in-patients accessed the day hospital and its activities even when they were confined to bed:

*There was a patient in an in-patient hospital bed facing towards the window but positioned so that she was part of the group. She had a portable oxygen bottle beside the bed. The sun was bathing her and she was facing towards the window. I (researcher) hadn’t met her so I introduced myself and explained why I was there. She told me that she was enjoying being in the day hospital as she hadn’t been out of the ward for some time and was “fed up looking at the four walls”.* (Study 2, palliative care day hospital, 03.08.11)

The extract shows that to some degree, interactions between in-patients and day hospital patients were purposeful, with the exchanges that resulted from these spatial entanglements exploited for the benefit of both groups of patients. Coming into contact with in-patients challenged patients attending day hospital to confront death. This was seen as positive by the specialist palliative care hospital team who operate in a paradigm of ‘open awareness’. Similarly, spending time in the day hospital could temporarily free in-patients from the confines of the ward and the informal set-up enhanced relations between the staff and patients.

In the palliative care context, the relational mode involved staff, patients and their carers engaging with one another affectively across public, private biomedical and administrative spaces that often extended beyond traditional sites of care.

## 5 Discussion

This paper has shown that two key modes of safety and wellbeing existed across UK primary care and Australian palliative care settings. The technical mode was driven by formal approaches to safety by staff members based on a singular view of the patient in terms of technical or biomedical need. Technical safety-wellbeing configurations were spatially controlled by healthcare staff across public, private, biomedical and administrative spaces. In contrast, the relational mode was primarily driven by the desire by staff to attend to the wellbeing of patients and their carers. This resulted in more informal approaches to safety being employed that relied on existing knowledge and informal communication, often across similar spaces to the technical mode (e.g. consulting rooms, reception areas, hospital wards).

While the technical and relational modes possessed similar characteristics across both contexts, important differences also existed. Within UK primary care, the technical mode was often employed by staff when pressure on time was high, with this in turn ordering both the biomedical and administrative spaces where patients received care and minimising staff focus on their wellbeing. In palliative care, formal safety rules were often followed if staff felt uncomfortable or unsupported when responding to specific patient requests. In primary care, the relational mode often involved staff and patients opportunistically carving out informal spaces in which to enhance their mutual wellbeing within the context of existing practice routines, spaces and relationships. In contrast, the relational mode in palliative care often took place in private spaces or in public spaces beyond traditional sites of care within more focused time periods. These differences reflect the unique pressures of each healthcare setting and the ability of people and spaces to adapt to these demands.

Our findings support previous studies of safety and space in healthcare organisational settings (Hor et al., 2014, Iedema et al., 2010). The paper also builds on this work by focusing attention on agency as distributed across people and the material world as a “capacity of social relationships” such that space is “what people do, not where they are” (Corsín-Jiménez, 2003: 140). Thus, rather than being site-defined, a spatial capacities lens has enabled us to examine how clinicians, patients and carers engage in both technical and relational modes of safety and wellbeing, often within the same spaces, that are dependent on the pressures that they are under and the opportunities available to them at particular times. Furthermore, rather than focusing primarily on inter-professional interactions, this approach widens the analytic lens to examine the relationship between clinicians, administrative staff, patients and carers. While the technical-bureaucratic safety mode employs a singular perspective on patients and carers in terms of very specific needs, the relational-wellbeing mode requires a more complex perspective on the patient as a ‘multiple’ (Mol, 2002) whole person with broader relational, emotional and spiritual needs. In the contemporary international context of increasing workloads and burnout, this paper has shown that it is possible for clinicians and non-clinicians to carve informal, holistic and relational approaches to safety within the same spaces as more technical ones, and that quality and safety should not be dependent on responsiveness to *either* efficiency *or* wellbeing.

The paper also extends extant research on safety and space to incorporate wellbeing as an integral dimension of patient safety. While recent studies have examined positive dimensions of safety (Hollnagel, 2014, Mesman, 2011), none have interlinked safety, wellbeing and space. This paper has drawn on more recent conceptualisations of wellbeing and place developed within anthropology that go beyond a location-oriented notion of space as “the context in which wellbeing as an outcome emerges” (Atkinson et al., 2012:, p.8) towards an understanding of wellbeing where place forms an integral part of a complex nexus of people, practices, emotions and memory (Ferraro and Sarmiento Barletti, 2016). This paper also contributes to this more recent body of research by bringing relational atunement between staff, patients and their carers in the achievement of wellbeing and its relationship with safety to the fore. In particular, it highlights the importance of affective, non-verbal elements of communication as key dimensions of everyday safety practices between these actors (Jerak-Zuiderent, 2012).

In a recent UK National Health Service (NHS) white paper, Bevan and Fairman (2014) write that effective patient safety change can only be realised through increased attention to tacit knowledge, emotional connections and relationships through “real-time, constantly-changing, collaborative support for learning in workplace situations” (p.14). Citing Jorm et al. (2009), the Australian Commission for Safety and Quality in Health Care similarly suggest refocusing the way that safety and quality in healthcare are approached, as “traditional models of patient safety are not patient centred enough to be used as a comprehensive approach to improving safety and quality” (Australian Commission for Safety and Quality in Health Care, 2010, p.51). Our findings support these views, with a more holistic, person-centred form of safety only achievable if it is reframed towards relational wellbeing as lived, socio-spatial practice.

While there are many potential benefits to attending to a relational-wellbeing mode of safety, focusing solely on this may also be problematic. This paper has shown that practicing safety frequently requires multiple trade-offs, with the technical mode of safety often necessary in situations where there are time pressures or highly technical tasks to perform. Notwithstanding such trade-offs, we contend that refocusing patient safety as much towards the relational mode as the technical one has important implications for policy and practice. When redesigned towards both safety and wellbeing, healthcare settings themselves would be recognised as clinical interventions rather than simply providing a container in which they occur. While some areas of healthcare such as midwifery (McCourt et al., 2012) are already paying increased attention to matters of wellbeing, we have shown that other areas also have the potential to become spaces that promote wellbeing. Ham and Berwick (2017), for example, argue for wards to be defined as “places for healing, recovery and care” where the “quality of relational care would have equal priority to clinical quality and patient safety, and changes in the physical environment, the conduct of staff and the organisation of care would follow” (p.76). Such an approach would mean that matters of patient safety would attend to the physical, social and emotional needs of staff, patients and carers. It would also not be site-defined but instead focus on patient and carers’ needs wherever they are cared for across primary, secondary or community healthcare settings.

## 6 Conclusions

Increasing workloads in healthcare internationally mean that policymakers and practitioners are under constant pressure to develop safe and efficient ways of delivering care (Hobbs et al., 2016, Thompson and Walter, 2016). At the same time, ageing patient populations mean that there is also pressure on practitioners to deliver high quality care that is responsive to increasingly complex patient needs, for example, regarding multimorbidity and polypharmacy (Guthrie et al., 2012). A key issue for policymakers and practitioners is thus to find novel methods and contexts through which to respond to these often competing challenges. Incorporating relational approaches to safety and wellbeing into existing technical ones across public, private, biomedical and administrative spaces means that greater attention can be paid to the wellbeing of both clinical and non-clinical staff as well as to patients and carers within the consulting room and beyond.
